# Circulating mitochondrial DNA-triggered autophagy dysfunction via STING underlies sepsis-related acute lung injury

**DOI:** 10.1038/s41419-021-03961-9

**Published:** 2021-07-03

**Authors:** Qinjie Liu, Jie Wu, Xufei Zhang, Xuanheng Li, Xiuwen Wu, Yun Zhao, Jianan Ren

**Affiliations:** 1 Research Institute of General Surgery, Jinling Hospital, Medical school of Nanjing University, Nanjing, China; 2 Department of General Surgery, The Affiliated BenQ Hospital of Nanjing Medical University, Nanjing, China; 3 Research Institute of General Surgery, Jinling Hospital, School of Medicine, Southeast University, Nanjing, China; 4 Research Institute of General Surgery, Jinling Hospital, Nanjing Medical University, Nanjing, China; 5 Research Institute of General Surgery, Jinling Hospital, Nanjing, China

**Keywords:** Infection, Sepsis

## Abstract

The STING pathway and its induction of autophagy initiate a potent immune defense response upon the recognition of pathogenic DNA. However, this protective response is minimal, as STING activation worsens organ damage, and abnormal autophagy is observed during progressive sepsis. Whether and how the STING pathway affects autophagic flux during sepsis-induced acute lung injury (sALI) are currently unknown. Here, we demonstrate that the level of circulating mtDNA and degree of STING activation are increased in sALI patients. Furthermore, STING activation was found to play a pivotal role in mtDNA-mediated lung injury by evoking an inflammatory storm and disturbing autophagy. Mechanistically, STING activation interferes with lysosomal acidification in an interferon (IFN)-dependent manner without affecting autophagosome biogenesis or fusion, aggravating sepsis. Induction of autophagy or STING deficiency alleviated lung injury. These findings provide new insights into the role of STING in the regulatory mechanisms behind extrapulmonary sALI.

## Introduction

Sepsis is a life-threatening condition and a major global health challenge [1]. The lung is the first organ to suffer from sepsis-induced insult, which results in acute lung injury (ALI) as well as its more severe form, acute respiratory distress syndrome (ARDS) [2]. During ICU hospitalization, the incidence of ARDS is approximately 40%, and despite modern medical care, therapies to prevent or treat lung injury in sepsis remain elusive [3]. Thus, it is critical to dissect the molecular mechanisms leading to sepsis-induced ALI (sALI) and the translational implications of these findings.

Damage-associated molecular patterns (DAMPs) released by stressed or injured cells and tissues were recently shown to play a vital role in the development of critical illnesses. Specifically, numerous clinical studies have confirmed that the level of circulating mitochondrial DNA (mtDNA) is closely related to the severity and prognosis of sepsis and ARDS [2]. In our previous studies, we have found that mtDNA plays a fundamental role in the mechanism of cellular injury and lethal sepsis by triggering the stimulator of interferon genes (STING) pathway, an intracellular DNA-sensing pattern recognition receptor, leading to remote organ injury [4, 5]. However, a translation gap between studies of ALI due to clinical nonpulmonary insult and preclinical mtDNA-mediated immune cascade studies remains, as the mechanism underlying the development of lung injury from infection at a distant site is multifactorial and has not been well addressed.

STING was initially recognized as an important molecule in immunity that detects DNA from infected pathogens or cytoplasmic DNA and triggers immune defense [6]. However, increasing evidence has indicated that STING pathway activation is a double-edged sword that can also cause tissue inflammation and destruction [7]. Overactivation of STING signaling or gain-of-function mutations in STING has been shown to contribute to systemic lupus erythematosus (SLE), STING-associated vasculopathy with onset in infancy (SAVI), and sepsis [8]. Interestingly, autophagy disruption has been observed under similar disease conditions [9]. Whether STING activation-mediated injury is associated with autophagic flux changes in sepsis and related organ injury remains unclear.

Here, we found that the level of circulating mtDNA and degree of STING activation was increased and that autophagic flux was changed in patients with sALI compared with sepsis patients. Furthermore, we explored the role of circulating mtDNA in extrapulmonary sepsis-induced lung injury. We show that STING activation plays pivotal role in mtDNA-mediated lung injury by evoking an inflammatory storm and disturbing normal autophagy. Mechanically, we also demonstrate that STING activation interferes with lysosomal acidification during autophagy in an interferon (IFN)-dependent manner to aggravate the disease. Our data thus provide evidence that circulating mtDNA-triggered autophagy dysfunction via activation of the STING pathway underlies sALI.

## Results

### Elevated levels of circulating mtDNA and STING activation are associated with disease severity in sALI patients

**Fig. 1:**
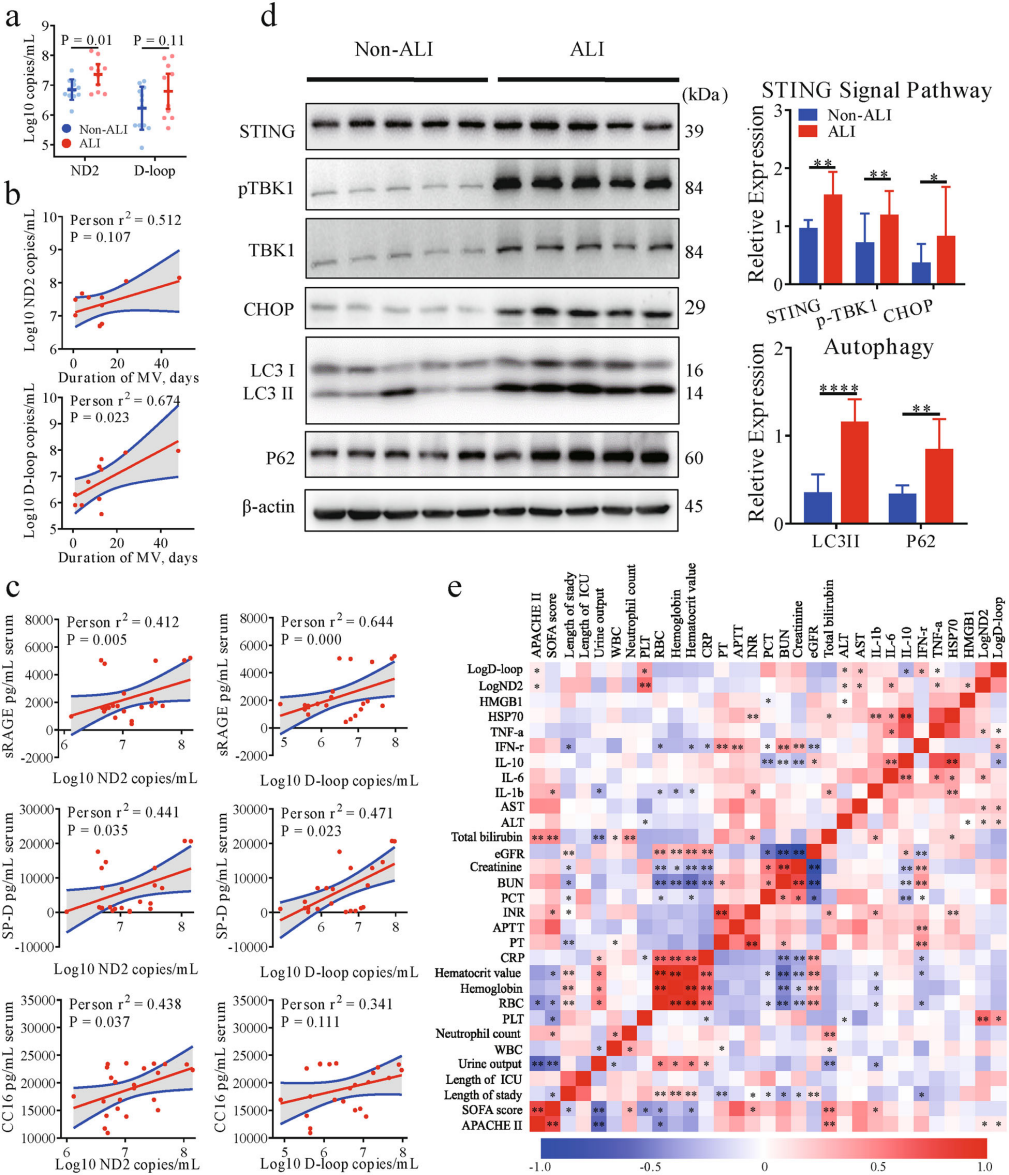
Elevated circulating levels of mtDNA and STING activation are associated with disease severity in septic patients. **a** mtDNA (ND2, D-loop) was measured in patients with sepsis with and without ALI. **b** The correlation between the serum level of mtDNA and duration of MV was assessed using the Spearman correlation test. **c** The correlations between the serum level of mtDNA and biomarkers of lung injury were assessed using the Spearman correlation test. **d** Western blot analysis of STING and autophagy signaling in PBMCs from patients with or without ALI. Two-tailed Student’s *t*-test was used to determine statistical significance, *p < 0.05; **p < 0.005; ***p < 0.0001. ns not significant. **e** Correlation matrix for circulating levels of mtDNA and blood biomarkers in critically ill patients.

Previous reports have indicated that circulating mtDNA can serve as an effective predictor of outcome in several chronic lung disease and sepsis [2, 10]. To further assess the relationship between mtDNA levels and sALI, we monitored the clinical characteristics, inflammatory indexes, and outcomes of sALI patients and sepsis patients without ALI. As expected, the level of circulating mtDNA was increased in sALI patients compared to septic patients without ALI and positively correlated with the clinical and experimental characteristics of the sALI patients, such as the duration of mechanical ventilation (MV), biomarkers of lung injury, circulatory inflammation and organ injury. These changes were accompanied by the activation of STING pathway signaling (Fig. 1b–d) in PBMCs from sALI patients.

In addition, we evaluated changes in expression of the autophagy protein LC3B and SQSTM1/p62 and found that both LC3-II conversion and the SQSTM1/p62 were increased in sALI patients compared to patients with sepsis but not ALI. Meanwhile, the expression of CHOP, an endoplasmic reticulum (ER) stress marker protein, was elevated in sALI patients (Fig. 1d). Similar results were also identified by analysis of transcriptomic data derived from the GSE66890 dataset [11], which showed that the level of cGAS and STING expression were increased in patients with sALI patients with sepsis (Fig. S1). Together, these results suggest that circulating mtDNA levels are associated with poor prognosis in sALI patients, which might account for the observed STING activation and abnormal autophagy.

### Circulating mtDNA enhances ALI by activating the STING pathway and impairing autophagy

**Fig. 2:**
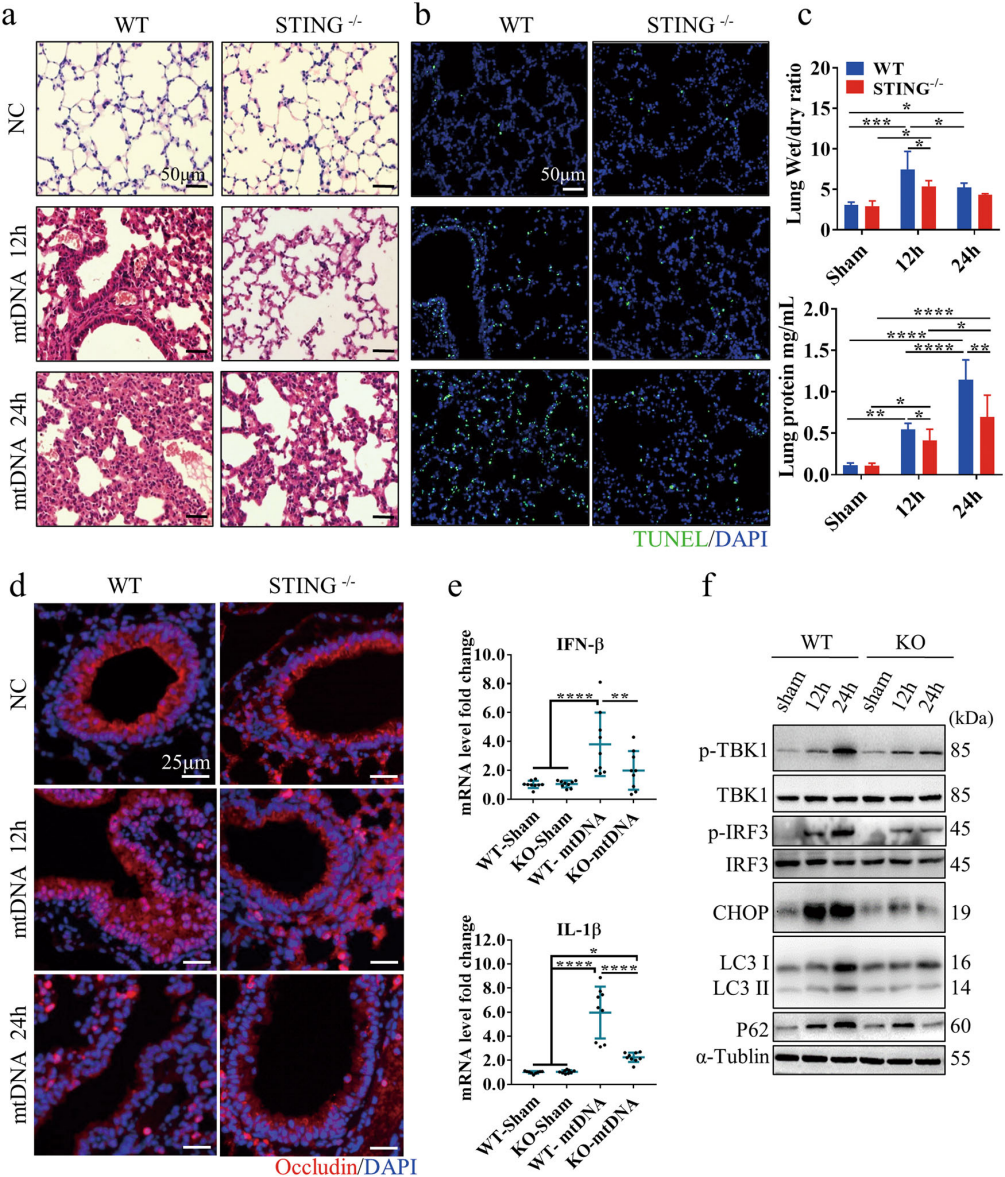
Circulating mtDNA contributes to ALI by activating the STING pathway and impairing autophagy. Representative images of lung H&E (**a**) and TUNEL staining (**b**) in WT and STING ^-/-^ mice at 12 or 24 h after mtDNA administration. The scale bar represents 50 μm. **c** The lung wet/dry ratio and BALF protein levels in WT and STING^-/-^ mice. **d** Immunofluorescence staining of occludin in the lung tissue of mice. Red, occludin immunostaining; blue, DNA stained with DAPI. The scale bar represents 25 μm. **e** qPCR analysis of IFN-β and IL-1β mRNA in the indicated tissues of mice at 24 h after mtDNA injection. **f** Western blot analysis of STING and autophagy signaling in the lungs of mice at 12 h and 24 h after intraperitoneal injection of mtDNA. Each panel shown represents the mean ± SD taken from at least three independent experiments. *p < 0.05; **p < 0.005; ***p < 0.0001. ns not significant. Two-tailed Student’s *t*-test was used to determine statistical significance.

We next investigated the role of circulating mtDNA in ALI by generation an intraperitoneal mtDNA injection model. No animal died due to mtDNA injection, but we observed pronounced lung injury at 24 h after mtDNA administration in the WT group (Fig. 2a). Moreover, The WT group exhibited a significantly higher proportion of dead cells with more severe lung edema and protein exudation than the STING^-/-^ group (Fig. 2b, c). Moreover, we analyzed tight junction protein expression and localization to evaluate the lung epithelial barrier after mtDNA injection. As shown in Fig. 2d, we observed that over time, the impairment of occludin expression and distribution induced by mtDNA administration increased in the WT group compared with the STING^-/-^ group. Consistently, the mRNA levels of IFN-β and IL-1β were significantly increased in WT mice compared with STING^-/-^ mice after mtDNA injection (Fig. 2e). We also found that STING pathway activation and ER stress injury, as assessed by measuring the expression of CHOP, were increased after mtDNA administration. Notably, LC3-II conversion and p62 accumulation were observed, especially after 24 h, in the WT mice but not the STING^-/-^ mice (Fig. 2f).

We next measured the mRNA levels of autophagy- and STING-related genes to identified the step in autophagic flux that is impaired in sALI and when circulating mtDNA levels are high. We found no difference in these mRNA levels between the WT and STING^-/-^ groups at the initiation of autophagy. Interestingly, the mRNA expression level of *p62*, a substrate of autophagy, was remarkably increased in the STING^-/-^ group (Fig. S2), but its protein expression level was lower in the STING^-/-^ group than in the WT group (Fig. 2f). These results indicate that increased circulating levels of mtDNA act as a key link in sALI because they activate the STING pathway and impair autophagy.

### mtDNA impairs the completion of autophagy in a manner dependent on STING signaling pathway activation

**Fig. 3:**
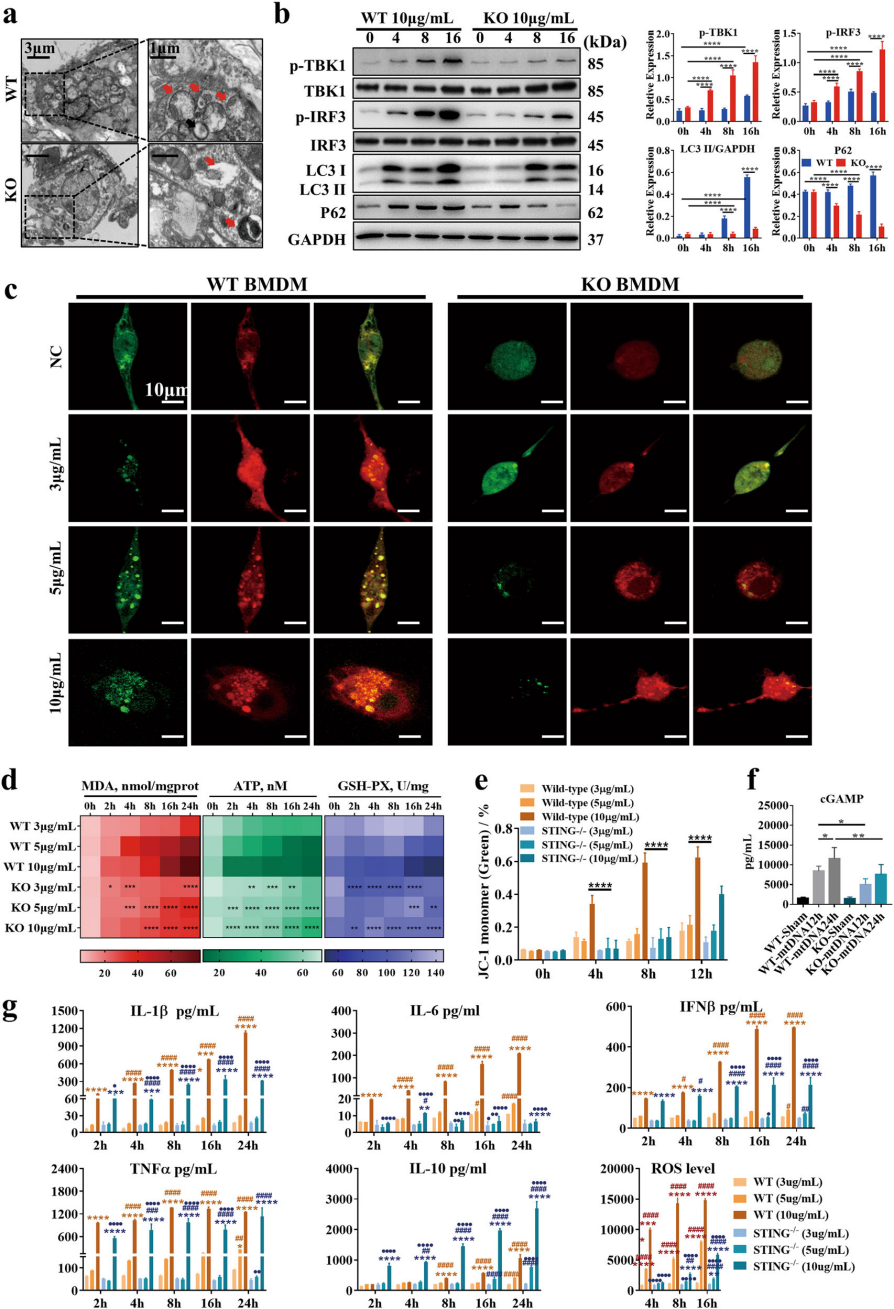
mtDNA impairs the completion of autophagy in a manner dependent on STING activation. **a** Representative electron microscopic images of autophagic vesicles in BALF macrophages from WT or knockout mice after stimulation with 10 μg/mL mtDNA. **b** Western blot analysis of STING and autophagy signaling in BMDMs after 0, 4, 8, and 16 h of stimulation with 10 μg/mL mtDNA. **c** Representative fluorescent images of BMDMs transfected with pMRX-IP-GFP-LC3-RFP-LC3ΔG and treated with 3, 5, or 10 μg/mL mtDNA. **d** MDA, ATP, and GSH-PX content of BMDMs after stimulation with 3, 5, and 10 μg/mL mtDNA for 2, 4, 8, 16, and 24 h. **e** The proportions of monomeric JC-1. **f** Quantification of intracellular cGAMP levels in BMDMs after stimulation with 10 μg/mL mtDNA for 12 and 24 h. Two-tailed Student’s *t*-test was used to determine statistical significance, *p < 0.05; **p < 0.005; ***p < 0.0001. ns not significant. **g** The levels of cytokines in the supernatant and intracellular ROS were assayed after stimulation with 3, 5, and 10 μg/mL mtDNA for 2, 4, 8, 16, and 24 h. Each panel shown represents the mean ± SD. •The STING^−/−^ group was compared with the WT group at the same time point and concentration, • < 0.05; •• < 0.01; ••• < 0.001; •••• < 0.0001; #stimulation with the same concentration of mtDNA, each time point was compared with 2 h in the same cell type, # < 0.05; ## < 0.01; ### < 0.001; #### < 0.0001; *At the same time, point, the effects of different concentrations of mtDNA were compared with those of the lowest concentration of mtDNA in the same cell type, * < 0.05; ** < 0.01; *** < 0.001; **** < 0.000. One-way ANOVA followed by Tukey’s multiple comparisons test was performed.

To further verify the effects of mtDNA on autophagy, we collected BALF-derived macrophages from mice treated with mtDNA and observed them by TEM. The WT group, but not the STING^-/-^ group, showed the obvious accumulation of autophagosomes (Fig. 3a). We next isolated BMDMs from WT and STING^-/-^ mice and stimulated them with 10 μg/mL mtDNA. In WT BMDMs, mtDNA treatment-induced STING pathway activation, LC3-II conversion and increased p62 expression in a time-dependent manner, but these effects were not observed in STING^-/-^ BMDMs (Fig. 3b). Because the increase in autophagy-associated protein accumulated could have been due to either increased autophagic flux or the blockade of autophagic flux, we used confocal analysis of tandem GFP-RFP-tagged LC3 fluorescence. The GFP-RFP probe can identify autophagosomes (GFP+/RFP+; yellow dots) and autolysosomes (GFP−/RFP+; red dots) because GFP fluorescence is quenched in compartments with a low pH. As shown in Fig. 3C, more yellow dots accumulated in WT BMDMs after stimulation with higher concentrations of mtDNA (5 μg/mL and 10 μg/mL mtDNA), while the number of red dots compared to yellow dots was increased in the STING^-/-^ BMDMs, indicating that autophagosomes or the abnormal lysosomal acidification of autolysosomes were significantly increased in the WT group compared to the STING^-/-^ group.

We estimated the levels of oxidative stress and inflammatory cytokines and the mitochondrial membrane potential (Fig. 3d–g, Fig. S3), which can indirectly reflect the blockade of autophagic flux damaging cell viability. The results showed a deeper color to represent more severe ROS injury, and time- and concentration-dependent mtDNA-induced oxidative injury in WT BMDMs was observed, but STING knockout blocked oxidative damage in cells by increasing the levels of GSH-Px and ATP and decreasing MDA production. Mitochondrial membrane potential was estimated, and the results showed that STING knockout in the mice could ameliorate mitochondrial injury, but ameliorated mitochondrial injury was not observed in the WT group (Fig. 3e, Fig. S3). Interestingly, we estimated the production of cGAMP in BMDMs stimulated with mtDNA and found that the level of cGAMP was remarkably higher in WT BMDMs than in STING^-/-^ BMDMs over time (Fig. 3f). This result indicates that mtDNA-mediated STING activation occurs in a vicious cycle that accelerates mitochondrial injury and produces more cGAMP. Moreover, we monitored changes in inflammation cytokine and ROS levels after mtDNA administration, which verified that STING knockout suppressed the production of inflammation cytokines and ROS after high-dose mtDNA stimulation, indicating the improvement of autophagy in the STING^-/-^ group (Fig. 3g).

mRNA expression levels in the STING pathway were increased in WT BMDMs stimulated with high-dose mtDNA, while ATG5 and p62 expression was higher in the STING^-/-^ group (Fig. S4A). We further verified the above results in RAW264.7 cells stimulated with DMXAA, and similar effects were observed, as shown in Fig. S4C, D. In addition, RAW264.7 cells were pretreated with RAP, a specific autophagy inducer, or 3-MA, which inhibits autophagy initiation, and then stimulated with DMXAA. The mRNA expression of inflammatory cytokines and STING pathway-related genes was decreased in the RAP pretreatment group but increased in the 3-MA group, which suggests that cellular injury induced by DMXAA could be improved by the induction of autophagy (Fig. S4B).

Taken together, these results confirmed that circulating mtDNA plays a crucial role in sALI by promoting the disruption of autophagic flux mediated by STING pathway activation.

### mtDNA-mediated STING activation impedes lysosomal acidification, aggravating disease

**Fig. 4:**
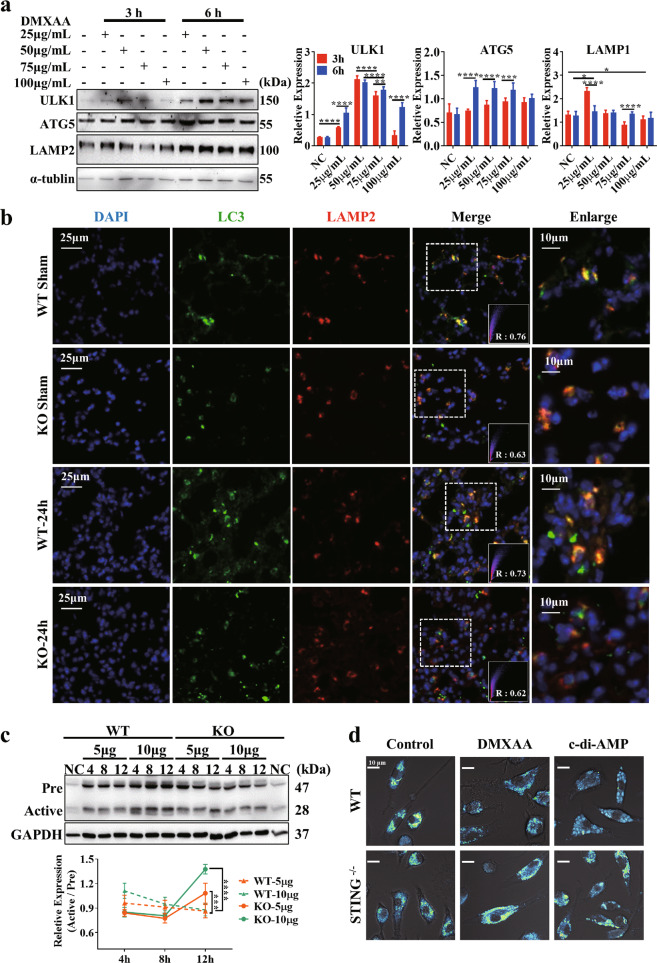
Activation of the STING pathway affects lysosomal acidification. **a** Western blot analysis of autophagy-related proteins in RAW264.7 cells after 3 and 6 h of stimulation with 25, 50, 75, and 100 μg/mL DMXAA. Two-tailed Student’s *t*-test was used to determine statistical significance, *p < 0.05; **p < 0.005; ***p < 0.0001. ns not significant. **b** Colocalization analysis of LC3 and LAMP2 in the lungs of mice after mtDNA administration. **c** Western blot analysis of cathepsin D in BMDMs at 4, 8, and 12 h after stimulation with 5 and 10 μg/mL mtDNA. One-way ANOVA followed by Tukey’s multiple comparisons test was performed. **d** Representative images showing a lysosomal pH probe in RAW264.7 cells. Cells were transfected with control siRNA or TBK1 siRNA 24 h before control or DMXAA treatment.

To identify the step in autophagy whose dysfunction is driven by the STING pathway, we further evaluated the initiation of autophagy, fusion of autophagosomes with lysosomes, and degradation of autophagosomal contents by proteases within the lysosome. Consistent with the above results, ATG5 protein expression was not affected by DMXAA stimulation. In addition, the levels of ULK1 and LAMP2 protein expression were even slightly increased at 6 h compared with those at 3 h upon DMXAA stimulation, which suggests that DMXAA-mediated STING activation does not affect the initiation of autophagy (Fig. 4a). IF staining of LC3 and LAMP2 was performed to assess the fusion of autophagosomes with lysosomes, and colocalization analysis indicated no difference in LC3 and LAMP2 colocalization between the lung tissues of WT and STING^-/-^ mice after mtDNA administration (Fig. 4b). Then, we assessed the degree of lysosomal acidification by observing changes in the activated cathepsin D levels after stimulation with mtDNA at different concentrations. Upon administration of mtDNA at a low concentration (3 μg/mL), the level of activated cathepsin D remained steady over time in both groups (Fig. S5A); however, the level of activated cathepsin D decreased over time in WT BMDMs upon administration of mtDNA at higher concentrations (5 and 10 μg/mL), but the opposite trend was observed in the KO group (Fig. 4c). Furthermore, we used the LysoSensor™ yellow/blue DND-160 probe to evaluate the lysosomal pH. LysoSensor dye produces blue fluorescence in neutral environments that changes to yellow fluorescence in more acidic environments. As shown in Fig. 4d, we treated BMDMs isolated from WT and STING^-/-^ mice with STING agonists and found that STING activation remarkably inhibited lysosomal acidification in WT BMDMs but not STING^-/-^ BMDMs. These data suggest that the STING activation-mediated defect in autophagic flux is dependent on disordered lysosomal acidification, which impedes cargo clearance.

To further elucidate which of two downstream signaling pathways, the TBK1 or NF-κB pathway, plays a more dominant role in the STING-mediated obstruction of autophagic flux, small interfering RNAs against TBK1 and NF-κB1 (p105) were used. Validation of the effects of transfection with the two siRNAs was performed by qPCR (Fig. S5B). Then, Baf was used for autophagic flux assays since the amount of LC3-II in the presence of Baf can reflect the basal autophagy level. As shown in Fig. S5C, D, we measured the ratio of LC3-II conversion in the presence of siRNAs with or without Baf in an unstimulated or a DMXAA-stimulated state. Autophagic flux was not improved in the presence of siNF-κB, but siTBK1 ameliorated the interruption of autophagic flux. Consistently, we observed increased lysosomal acidification upon TBK1 interference, but NF-κB interference did not clearly enhance lysosomal acidification. Collectively, these results suggest that STING-mediated autophagic flux blockade is partially associated with TBK1 downstream signaling.

### STING activation accompanied by autophagy deficiency is essential for lung injury in lethal sepsis models

**Fig. 5:**
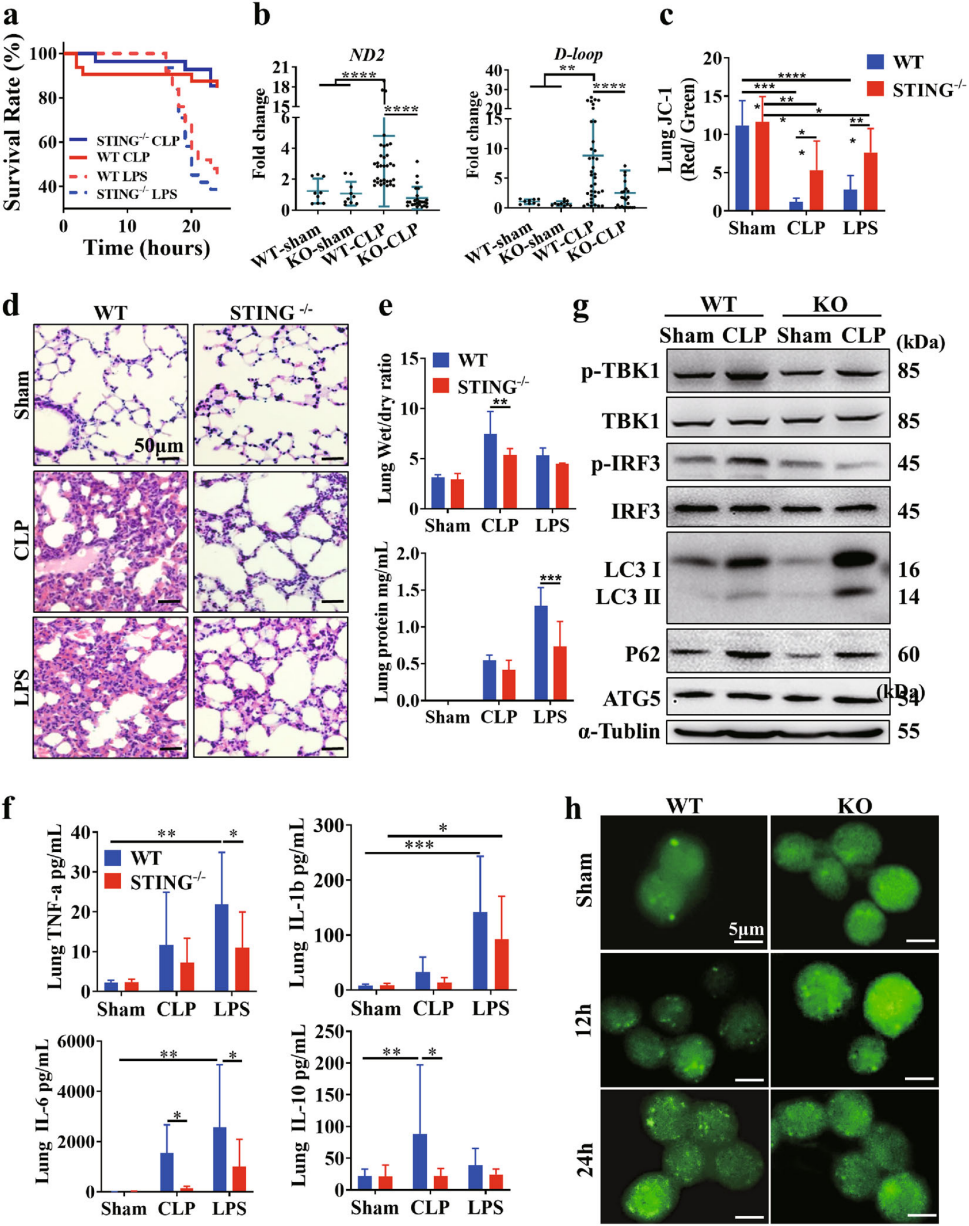
STING-dependent autophagy deficiency mediates lung damage in lethal sepsis models. **a** Mice with the indicated genotypes were subjected to CLP or LPS administration, and animal survival was monitored (*n* = 40mice/group; *p < 0.05, Kaplan–Meier survival analysis). **b** Serum mtDNA (*ND2* and *D-loop*) levels at 24 h after CLP in WT and STING^-/-^ mice. **c** The ratio of JC-1 monomer/aggregate. **d** Representative images of lung H&E staining in WT and STING^-/-^ mice at 24 h after CLP or LPS administration. The scale bar represents 50 μm. **e** The lung wet/dry ratio and BALF protein level in WT and STING^-/-^ mice at 24 h after CLP or LPS administration. **f** Levels of TNF-a, IL-1b, IL-6, and IL-10in the lung at 24 h after CLP or LPS administration. **g** Western blot analysis of the STING pathway and autophagy-related proteins in the lungs of WT and STING^-/-^ mice at 24 h after CLP. **h** Representative fluorescent images showing LC3 in alveolar macrophages. Two-tailed Student’s *t*-test was used to determine statistical significance, *p < 0.05; **p < 0.005; ***p < 0.0001. ns not significant.

To address the role of STING activation in sepsis with ALI, we administered LPS injections or carried out CLP to mimic the human sepsis with ALI in STING^-/-^ and WT mice. Survival analysis showed that STING blockade significantly improved survival in sepsis models (Fig. 5a). Consistent with the data from clinical samples, the levels of circulating mtDNA were elevated in the WT group, but this increase was inhibited by STING knockout (Fig. 5b). Meanwhile, the severity of lung injury and mitochondrial membrane potential was significantly ameliorated in the STING^-/-^ group (Fig. 5c). Moreover, lung tissue inflammatory factors, the BALF protein concentration, and the lung wet/dry ratio were were significantly improved in the STING^-/-^ mice than in WT group(Fig. 5d–f). We also found that both the conversion of LC3-II and level of p62 were increased at 24 h after modeling, which was similar to observations in sALI patients (Fig. 5g). Moreover, the level of p62 in the WT model group was higher than that in the STING^-/-^ model group, and increased LC3 puncta were observed in alveolar macrophages from WT model mice (Fig. 5h), whereas ATG5 expression remained unchanged (Fig. 5g).

These results indicate that autophagic flux was blunted after excess STING activation. In line with the characteristics of lung injury, an excessive systemic inflammatory response was observed in the WT model group, but this observation was markedly improved in the STING^-/-^ model group (Fig. S6).

## Discussion

Trauma and sepsis contribute to >50% of ARDS cases [12], but not all of these cases can be accounted for by viral or bacterial infections that damage the lung. Since the first clinical trials to investigate the relationship between mtDNA and critical illness in 2013 [13], an increasing number of studies have focused on its diagnostic and predictive performance in diverse diseases. A recent cohort study of 350 trauma and sepsis patients first show a significant link between circulating mtDNA and ARDS [2]. However, the mechanism by which nonpulmonary insults precipitate lung injury needs to be explained.

**Fig. 6:**
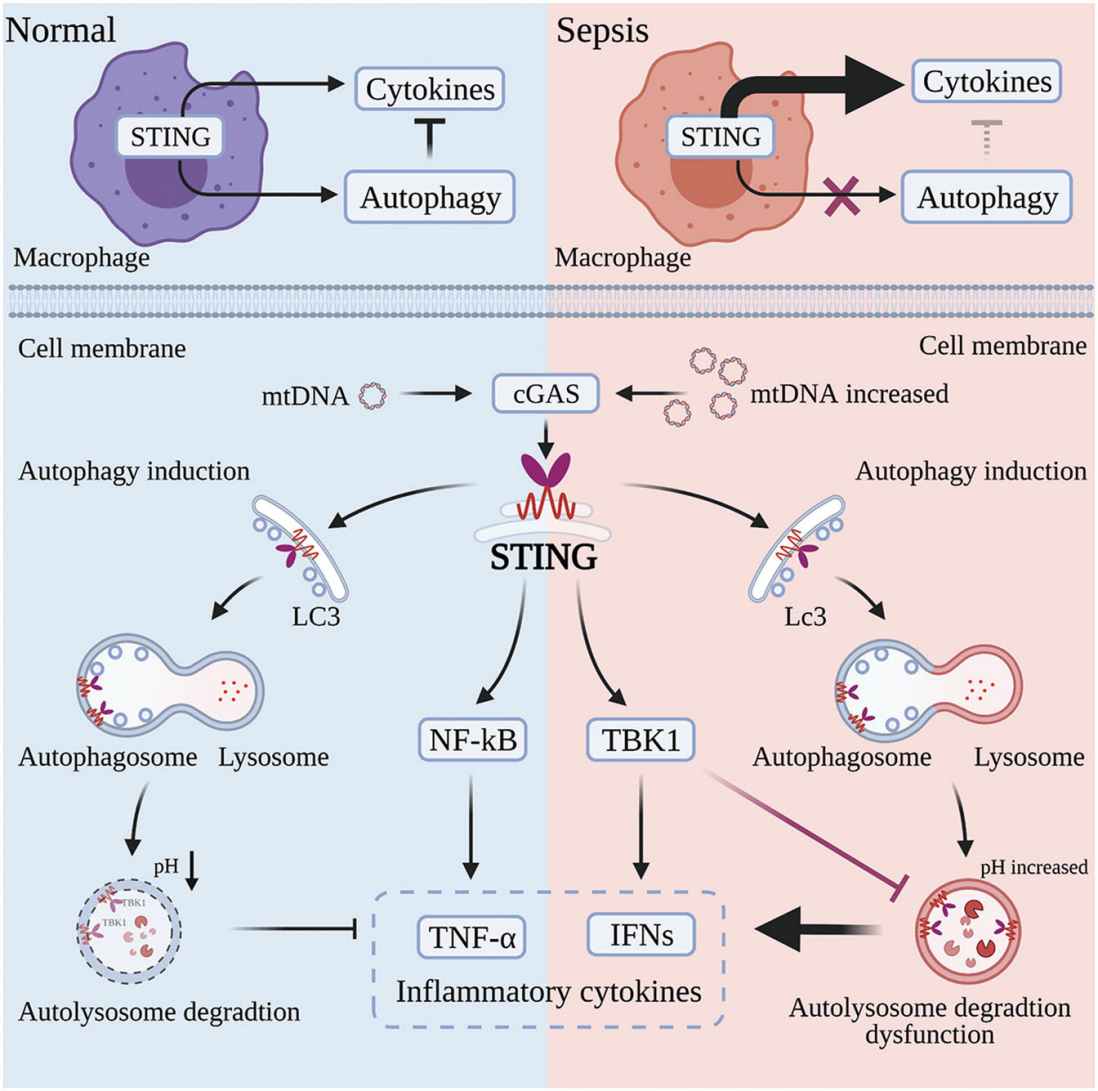
Schematic representation of circulating mtDNA-mediated acute lung injury during sepsis due to STING-dependent autophagy dysfunction. Infection and stress facilitate mtDNA release from injured cells into the circulation, and this mtDNA is recognized by macrophages. Excess activation of the STING pathway leads to autophagic flux blockade by affecting lysosomal acidification in a TBK1-dependent manner, resulting in a vicious cycle and remote organ injury. Created with BioRender.com.

In the present study, we found that circulating mtDNA is significantly associated with the incidence of ALI and severity of disease in patients with sepsis from a nonpulmonary source. Furthermore, our results first indicated that the critical detrimental effects of circulating mtDNA on ALI occur mainly through the STING pathway, and these effects were significantly improved by STING knockout in sepsis models and an mtDNA administration model. The most intriguing finding of this study is that circulating mtDNA-mediated ALI in sepsis from a nonpulmonary source results from excess STING activation-induced dysfunction of lysosomal acidification. This new finding is illustrated in Fig. 6.

The cGAS-STING pathway is a major pathway that mediates the initial immune defense against infections by diverse classes of pathogens that contain DNA or generate DNA in their life cycles [6], including DNA viruses, bacteria, and parasites [14]. However, the role of the STING pathway is not limited to antimicrobial defense. In response to cellular stress, mtDNA enters the cytoplasm, where it activates cGAS-STING to trigger inflammatory responses [15, 16]. A prime example of this process can be seen in patients with gain-of-function mutations in STING who suffer severe systemic inflammation, skin vasculopathy, broad interstitial lung disease, and recurrent bacterial infection, collectively termed STING-associated vasculopathy with onset in infancy (SAVI) [8]. The cGAS-STING pathway has also been linked to lung injury and inflammation. Deficiency in cGAS or STING ameliorates silica-induced lung inflammation [17]. Self-DNA released upon cigarette smoke exposure was found to activate cGAS-STING signaling, leading to type-I IFN-dependent lung inflammation [18]. Recently, Copaescu et al. reported that self-DNA release after SARS-CoV-2 infection could activate the STING pathway, producing excessive type-I IFN and inflammatory cytokines and contributing to the severity of COVID-19 [19]. Therefore, STING may play distinct roles during the different stages of sepsis, and further exploration of this mechanism is needed.

Our findings first demonstrated that robust STING activation in macrophages by circulating mtDNA acts as an executor of local infection that leads to remote organ damage, and ALI during the development of sepsis. STING activation-induced changes in autophagy played a detrimental role in sALI, beyond its role as a negative regulator of inflammation. Thus, the interaction between STING and autophagy and changes in autophagic flux may explain the dual effects of STING in pathogen infection and sepsis.

Autophagy is a process that includes autophagosomal formation; autophagosome-lysosome fusion; and the delivery of soluble macromolecules, organelles, and other cytoplasmic contents to lysosomes for degradation. Autophagy has long been considered a cellular adaptive and protective biological process that limits cell damage and apoptosis in sepsis [20]. During the early stage of sepsis, autophagy protects the host from MODS by preventing the death of immune cells, maintaining homeostasis between the production of pro- and anti-inflammatory cytokines, and eliminating microbial infection [21]. A vast number of studies have uncovered the mechanism of STING-induced autophagy for the elimination of intracellular microorganisms and subcellular organelles [22]. cGAS-STING-mediated autophagy is also known to be indispensable for removing cytosolic DNA and inflammatory signaling factors to restrict the inflammatory response raised by the pathway itself under physiological or compensable disease conditions [23].

However, the benefit of autophagy is limited. Sepsis triggers autophagy at the initial stage of disease development, but autophagic flux decreases with the advancement of sepsis [24]. Recent reports show that autophagy increased proportionally to the magnitude of the challenge in models of mild sepsis, while this capability declined proportionally to the severity of the challenge under conditions of severe sepsis [25]. Moreover, disturbed autophagic processes in T cells were found in the later stages of sepsis, which was considered a major cause of immune suppression [26]. Thus, it is necessary to explore which part of autophagy is aberrant and why the relationship between autophagy turbulence and STING pathway activation in sepsis runs counter to this existing understanding.

Recent attention has been given to the adverse effects of STING pathway activation on autophagy and cell survival in inflammatory or autoimmune diseases. In traumatic brain injury, upregulated STING expression leads to increased expression of proinflammatory cytokines and reduced autophagy [27]. In addition, a study confirmed that in SLE, a representative type-I interferonopathy, IFNα impairs autophagic degradation of mtDNA, promoting STING activation [28]. In contrast, another study uncovered that IFN-I–dependent lysosome acidification in intestinal epithelial cells was associated with elevated *Salmonella enterica* serovar Typhimurium virulence gene expression and exacerbated cell death by controlling pH, and protease activity of lysosomes [29]. Thus, the underlying mechanism of the interaction between the STING pathway and autophagy during sepsis remains to be elucidated.

Our data reveal that STING signaling leads to the deceleration of lysosomal digestion by affecting lysosomal pH regulation, which is partially dependent on downstream TBK1 and not accompanied by an effect on the initial or fusion stage of autophagosomes. Indeed, STING-mediated autophagic flux blockade did not occur at the beginning of autophagy. Conversely, the activation of STING simultaneously induced autophagy even at low concentrations of mtDNA or at the initial stage during mtDNA stimulation. However, continuous STING pathway activation contributed to terminating autophagic flux by perturbing lysosomal digestion, which could be ameliorated by inducing autophagy or impeding the STING pathway (Fig. 3, Figs. S3, S4). In accordance with our previous study, STING activation was accompanied by overtly promoted lipid peroxidation injury, which also suggested that dampened autophagic flux occurred during this period [5]. Thus, the overwhelming STING activation-mediated changes in autophagic flux are a key turning point for poor clinical prognosis.

Abnormal autophagy mediated by STING might be a pivotal change. One study demonstrated that telomeric damage results in the release of DNA fragments into the cytoplasm, which triggers STING-induced autophagy and is critical for cell death [30]. Another recent study reported that *Burkholderia pseudomallei* infection-mediated host cell fusion could trigger autophagic death by activating the cGAS-STING pathway to limit aberrant cell division and cellular transformation [31]. Moreover, studies have found that STING activation results in pyroptosis by enhancing permeabilization of the lysosome membrane [32], leading to the necroptosis of tumor cells due to type-I IFN and TNFα production [33]. However, the mechanism of excess STING activation-induced adverse effects remains to be further investigated, as to whether these injuries caused by STING activation are tissue- or cell-specific, where the precise turning point at which the effect of STING activation switch from defense to damage, and what the biological functions of STING-induced autophagy changes in sepsis remain unclear.

In conclusion, our findings advance the understanding of sALI pathogenesis and unveil a previously unclear role for STING in obstructing sepsis-related autophagic flux. We highlight unique features of circulating mtDNA that are recognized by the STING pathway of macrophages, leading to lysosomal acidification dysfunction, and show that impeding autophagic flux is another mechanism of distant organ injury in sepsis that occurs in parallel with infectious injury caused by pathogenic bacteria. These findings provide important clues for developing novel sALI treatments targeting mtDNA or the STING pathway.

## Materials and methods

### Antibodies and reagents

Antibodies against the following were used: STING (13647, Cell Signaling Technology, Danvers, MA, USA); p-TBK1 (S172, 5483, Cell Signaling Technology, Danvers, MA, USA); TBK1 (38066, Cell Signaling Technology, Danvers, MA, USA); p-IRF3 (Ser396, 29047, Cell Signaling Technology, Danvers, MA, USA); IRF3 (4302, Cell Signaling Technology, Danvers, MA, USA); LC3A/B (12741, Cell Signaling Technology, Danvers, MA, USA); CHOP (2895, Cell Signaling Technology, Danvers, MA, USA); SQSTM1/p62 (39749, Cell Signaling Technology, Danvers, MA, USA); ULK1 (8054, Cell Signaling Technology, Danvers, MA, USA); Atg5 (12994, Cell Signaling Technology, Danvers, MA, USA); LAMP2 (ab13524, Abcam, UK); Cathepsin D (ab75852, Abcam, UK); β-actin (AC026, ABclonal, China); α-Tubulin (A6830, ABclonal, China); and ZO1 (ab221547, Abcam, UK). Also used in the experiments were Alexa Fluor® 555 anti-mouse IgG (ab150114, Abcam, UK), Alexa Fluor® 488 anti-rabbit IgG (ab150077, Abcam, UK), and Alexa Fluor® 633 anti-rat IgG (A-21094, Invitrogen, San Diego, CA, USA). Antibodies were diluted according to the manufacturer’s instructions. The following reagents were used: DMXAA (HY-10964, MedChemExpress, Princeton, NJ, USA), rapamycin (RAP) (HY-10219, MedChemExpress, Princeton, NJ, USA), 3-methyladenine (3-MA) (HY-19312, MedChemExpress, Princeton, NJ, USA), and bafilomycin A1 (Baf) (HY-100558, MedChemExpress, Princeton, NJ, USA).

### Identification of sepsis and ARDS

The diagnosis of ARDS was made according to the Berlin ARDS definition [34] based on the following criteria: (1) the presence of acute hypoxemic respiratory failure; (2) onset within 1 week of insult or the presence of new (within seven days) or worsening respiratory symptoms; (3) bilateral opacities on chest X-ray or computed tomography not fully explained by effusions, lobar or lung collapse, or nodules; and (4) cardiac failure that was not the primary cause of acute hypoxemic respiratory failure.

Sepsis and septic shock were defined according to the Third International Consensus Definitions for Sepsis and Septic Shock [35].

### Study population

The following criterion was used for eligibility: fulfill the criteria for a diagnosis of sepsis and/or ARDS. The following exclusion criteria were used: refused consent, history of chronic lung disease, ventilator-associated pneumonia, or younger than 18 years in age. Baseline demographic and clinical data were obtained from hospital records, which were automatically recorded by software or physicians. Gender, age, and clinical patient records are listed in Table S1.

### Peripheral blood mononuclear cell (PBMC) isolation

In brief, 3 ml of Lymphoprep (07801, STEMCELL technologies, Vancouver, BC, Canada) was added to a 15-ml SepMate^TM^ tube (85415, STEMCELL technologies, Vancouver, BC, Canada). Two milliliters of blood was diluted by mixing with 2 ml of phosphate-buffered saline (PBS), and the blood was overlaid on the Lymphoprep^TM^. Next, the mixture was centrifuged at 800 × g for 20 min at room temperature (RT) without braking. The upper plasma layer was removed, and the PBMC layer in the plasma was retained.

### Mice

Wild-type (WT) and STING^–/–^ C57BL/6 mice were purchased from the Nanjing Biomedical Research Institute of Nanjing University. All mice were on a C57BL/6 genetic background. Genotyping of the knockout mice was performed by PCR using DNA from the tail. All mice were two to three months old. The animals were randomly included in experimental groups. No animals were excluded because of illness after the experiments, and the animal experiments were carried out in a blinded fashion.

### Cecal ligation and puncture (CLP) model

The CLP method was used to generate a model of abdominal sepsis as described previously [36]. Briefly, mice were anesthetized with a mixture of intraperitoneal xylazine (50 mg/kg) and ketamine (50 mg/kg). A longitudinal incision was made to the middle of the abdomen, and the cecum was exposed. The cecum was ligated with a 5.0 monofilament suture just distal to the ileocecal junction (with more than 90% of the cecum ligated) and punctured with an 18-gauge needle. A small amount of feces was gently squeezed out of the perforation to ensure patency of puncture. The cecum was then placed back into the peritoneal cavity, and the incision was closed. In sham surgical controls, the cecum was exposed but not ligated or punctured and then returned to the abdominal cavity. All mice received 0.5 ml of PBS immediately after surgery for fluid resuscitation. At the end of the study, the mice were sacrificed, and bronchoalveolar lavage fluid (BALF), peritoneal lavage fluid (PLF), blood, and tissue were collected under sterile conditions.

### LPS model

Lipopolysaccharide (LPS) (L2630, Sigma-Aldrich, Germany) was given systemically with a single intraperitoneal injection of 10 mg/kg LPS to generate a model of systemic shock. Control mice received an intraperitoneal injection of PBS. The mice were sacrificed at 12- and 24-h following exposure.

### mtDNA administration

C57BL/6 mice were used for the intraperitoneal mtDNA (5 mg/kg) administration experiment. The selected DNA concentration was sufficient to induce systemic inflammatory response syndrome (SIRS) according to previous publications [36]. mtDNA was isolated from the livers of C57BL/6 mice subjected to ischemia and reperfusion using an mtDNA isolation kit (ab65321, Abcam, UK) following the manufacturer’s instructions. The DNA concentrations and purity were analyzed using a NanoDrop 2000 instrument (Thermo Fisher Scientific, Waltham, MA, USA). Then, we confirmed the absence of detectable endotoxin levels in the samples (Associates of Cape Cod Inc., East Falmouth, MA, USA). To further ensure the purity of the mtDNA, qPCR was conducted, and the nuclear DNA (nDNA) content in the isolated mtDNA samples was less than 0.1%.

### Mouse tissue collection and processing

Mice were euthanized via CO_2_ asphyxiation. After the instruments were rinsed with ethanol, the hilum of the left lung was ligated and removed for histologic studies. BALF was collected by flushing the right lung, after which alveolar swelling was observed, and the liquid was recovered after 15–30 s. The same operation was performed twice. The recovered lavage was centrifuged at 1000 × g and 4 °C for 5 min. Supernatants were collected and frozen for later analysis. Total protein in the BALF was quantified using the bicinchoninic acid (BCA) protein assay kit (P0012, Beyotime, China). Pelleted cells were resuspended in DMEM with 10% FBS, counted in a hemocytometer, and saved for future experiments.

PLF was collected 30 s after the intraperitoneal injection of 5 ml of PBS. The recovered lavage liquid was placed on ice and centrifuged at 1000 × g for 5 min, and the recovered supernatant was placed at −80 °C. The turbid sediments at the bottom, which were peritoneal macrophages, were preserved for subsequent cytokine detection.

### Macrophage culture, transfer, and treatments

Bone marrow-derived macrophages (BMDMs) were isolated and cultured as described. To generate BMDMs, marrow cells were differentiated in medium containing 10 ng/ml CSF-1 (315-03, PeproTech, Cranbury, NJ, USA) for 7 d. For further experiments, the cells were then cultured in medium containing gentamicin, 100 IU/ml penicillin, and 100 mg/ml streptomycin and stimulated with mtDNA or the appropriate drug, after which RNA and protein were collected 4, 8, or 16 h later. To prepare alveolar macrophages, cells in BALF (98% macrophages) from four groups of four naive wild-type mice per group were pooled to conduct further measurements.

### RNA isolation and real-time quantitative PCR

Circulating DNA was isolated from 200 μL of plasma using the QIAamp DNA Blood Mini Kit (Qiagen, Germany), and total RNA was isolated from lung tissue with TRIzol reagent (Life Technologies, Pittsburgh, PA, USA) according to the manufacturer’s instructions. The collected DNA was eluted in 100 μL of supplied buffer as previously described [37]. Mitochondrial genes (*D-loop* and *ND*_*2*_) were used to quantify cell-free mtDNA. No homology between mtDNA primers and bacterial DNA was found. All reactions were more than 98% efficient. All the experiments were performed with triplicate samples. The standard curve for the quantitative assay was assessed with serially diluted template cloned into plasmid DNA.

### Enzyme-linked immunosorbent assay

Concentrations of cytokines and markers of lung injury in tissues, plasma, BALF, and PLF were determined by enzyme-linked immunosorbent assay (eBioscience, San Diego, CA, USA) according to each manufacturer’s instructions. Intracellular cGAMP was quantified according to the manufacturer’s protocol (Arbo Assays, Ann Arbor, Michigan, USA).

### Western blotting

Cell lysates were collected from tissues in RIPA lysis buffer containing proteinase inhibitors. Protein concentrations were quantitated using a BCA protein assay kit (P0012, Beyotime, China), and samples containing 25 µg of protein each were mixed with loading buffer and boiled for 10 min at 95 °C. Proteins were separated by sodium dodecyl sulfate-polyacrylamide gel electrophoresis (SDS-PAGE) and transferred onto polyvinylidene fluoride (PVDF) membranes (Millipore, Billerica, MA, USA). After blocking for 1 h at RT, the membranes were then incubated with primary antibodies overnight at 4 °C. Then, the membranes were washed with 1X Tris-buffered saline and 0.1% Tween® 20 detergent (TBST) and incubated with secondary antibody conjugated to horseradish peroxidase (HRP) for 1 h at RT. The blots were imaged using a Tanon 5200 chemiluminescent imaging system (Tanon, Shanghai, China) and quantified with ImageJ software version 1.49 (National Institutes of Health, Bethesda, MD, USA).

### Immunofluorescence (IF) and immunohistochemistry (IHC)

For IF microscopy, cells were plated on poly-L-lysine-coated coverslips. When the appropriate treatments had been administered, the coverslips were washed once with PBS, fixed with 4% paraformaldehyde (PFA) for 20 min (RT), and permeabilized with 100% ice-cold MetOH for 10 min. The coverslips were blocked with 5% BSA for 1 h and stained overnight with primary antibodies at 4 °C. The slides were washed several times with PBS and incubated with the following secondary antibodies for 1 h at RT: Alexa Fluor® 555 anti-mouse IgG (ab150114, Abcam, UK), Alexa Fluor® 488 anti-rabbit IgG (ab150077, Abcam, UK), and Alexa Fluor® 633 anti-rat IgG (A-21094, Invitrogen, San Diego, CA, USA). For visualization of the nuclei, DAPI (28718-90-3, Sigma-Aldrich, Germany) was used (if needed).

For LysoSensor DND-160 (L7545, Invitrogen, San Diego, CA, USA) staining, the dye was added to the culture as suggested by the manufacturer, after which the samples were washed and mounted for immediate observation under confocal microscopy.

For IHC, sample sections were deparaffinized through a series of xylene baths, after which antigens were retrieved by steam treatment in 10 mM citrate buffer., The sections were then blocked with 3% hydrogen peroxide for 15 min at 37 °C, preincubated with blocking serum solution for 30 min at 37 °C, and then incubated at 4 °C with primary antibodies overnight. Subsequently, secondary antibodies were applied, and the nuclei were counterstained with hematoxylin.

Terminal deoxynucleotidyl transferase-mediated dUTP nick-end labeling (TUNEL) staining was performed using a commercial kit (KGA702, KeyGEN, China) according to the manufacturer’s instructions.

### Transmission electron microscopy (TEM)

After cells were collected, they were centrifuged, and the pellets were fixed with 0.1 M cacodylate buffer with a pH of 7.4 at RT. Sections were then processed by the UAM Electron Microscopy unit per standard protocols. Pictures were taken with a JEM-1010 transmission electron microscope (JEOL, USA).

### pMRX-IP-GFP-LC3-RFP-LC3ΔG assay

The pMRX-IP-GFP-LC3-RFP-LC3ΔG plasmid was a gift from Noboru Mizushima (Addgene plasmid # 84572; http://n2t.net/addgene:84572; RRID: Addgene_84572) [38]. Cultured cells seeded in six-well plates with microscope cover glasses were transfected for 24 h with pMRX-IP-GFP-LC3-RFP-LC3ΔG using INTERFERin (409-10, Polyplus, New York, NY, USA). The cells were treated with Sal as indicated in the figure legends. All cell images were obtained using an LSM880 confocal microscope (Carl Zeiss GmbH, Oberkochen, Germany). Autophagy was then measured by counting cells in GFP-LC3 puncta or GFP+/RFP+(yellow) and GFP-/RFP-LC3+(red) puncta with confocal microscope.

### Measurement of mitochondrial membrane potential

Mitochondrial membrane potential was assessed using the JC-1 assay kit (Beyotime Biotech, China) according to the manufacturer’s instructions. Briefly, cells digested from lung tissue were stained for 20 min at 37 °C in the dark and washed twice with the buffer provided with the kit. Then, the cells were analyzed using a FACSCalibur flow cytometer (Becton Dickinson, San Jose, California, USA) with excitation at 488 nm and emission at 530 nm.

### Measurement of the intracellular ATP content

The intracellular ATP content was measured using the ATP bioluminescence assay kit (Beyotime Biotech, China) according to the manufacturer’s instructions.

### Statistical analyses

Data are presented as the mean ± SEM unless otherwise indicated. Comparisons of groups were performed using Student’s *t*-test for repeated measurements to determine the levels of significance for differences between each group. For survival curve analysis, the survival status of the mice was observed every 12 h after CLP surgery, and the natural time of death after initiation of CLP was recorded. Data from multiple groups were analyzed with one-way ANOVA and Tukey’s posttest. A minimum of three independent experiments were performed, and differences for which *p* < 0.05 were considered statistically significant. Significance was determined using appropriate tests in GraphPad Prism, with *P* > 0.05 indicating nonsignificance (*p < 0.05, **p < 0.01, ***p < 0.001, ****p < 0.0001).

## Supplementary information

Supplemental material

## Data Availability

The data that support the findings of this study are available from the corresponding author upon reasonable request.
