# Supplementary material for: Circulating mitochondrial DNA-triggered autophagy dysfunction via STING underlies sepsis-related acute lung injury
Source: Cell Death Dis. 2021 Jul 3;12(7):673. doi: 10.1038/s41419-021-03961-9 (PMC8254453; doi:10.1038/s41419-021-03961-9)
Supplement: Supplementary file 1 — Supplemental material [file 41419_2021_3961_MOESM1_ESM.docx]

Supporting information for:

**Circulating mitochondrial DNA-triggered autophagy dysfunction via STING underlies sepsis-related acute lung injury**

**TABLE OF CONTENTS**

**SUPPLEMENTARY FIGURES……………………………………………………… 2**

**SUPPLEMENTARY TABLES……………………………………………………….. 9**

# SUPPLEMENTARY FIGURES


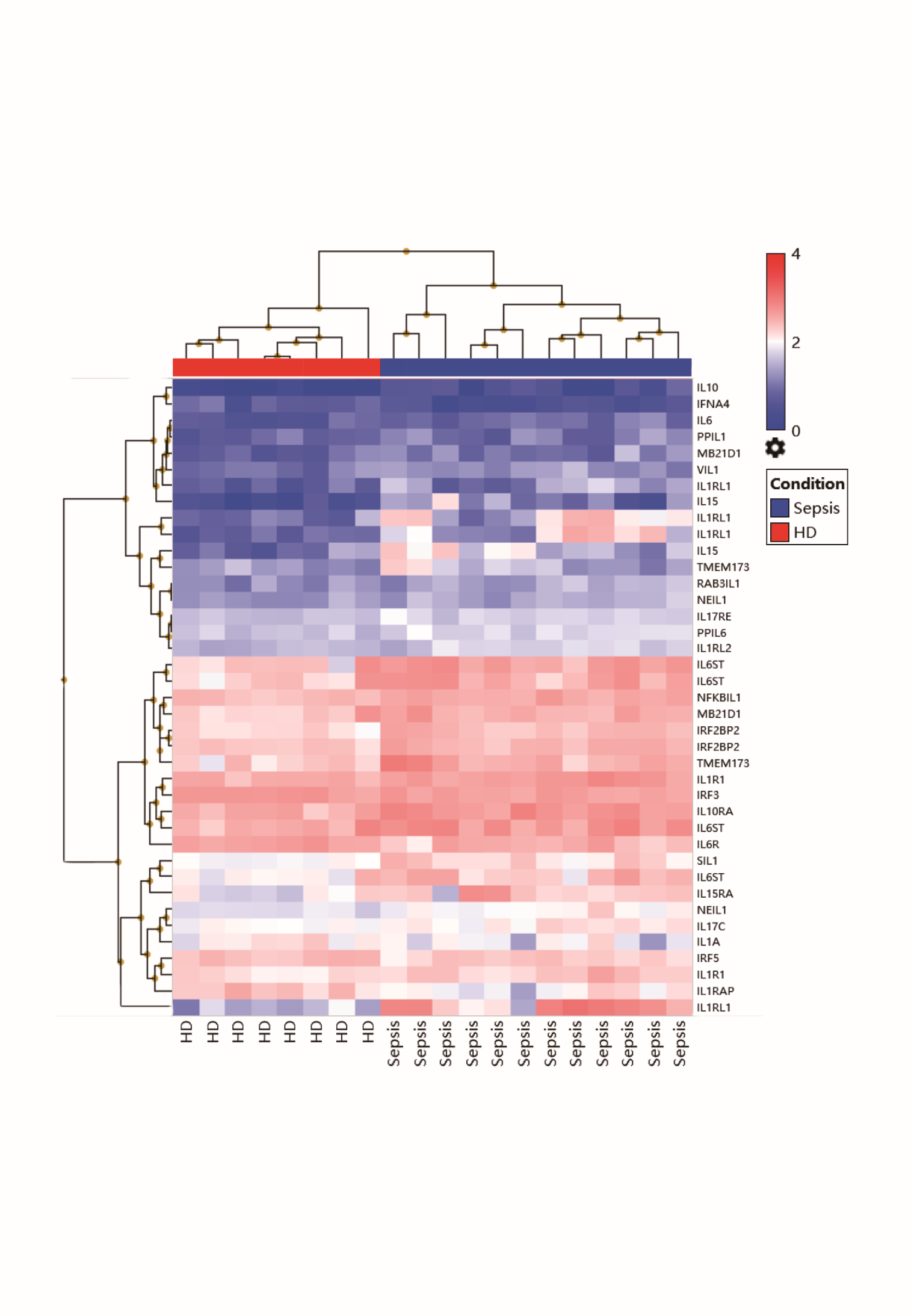


Figure S1. STING expression were increased in patients with sALI than in sepsis group. Transcriptomic data derived from the GSE66890 database. Analyzed with Transcriptome Analysis Console (TAC) Software (Affymetrix).


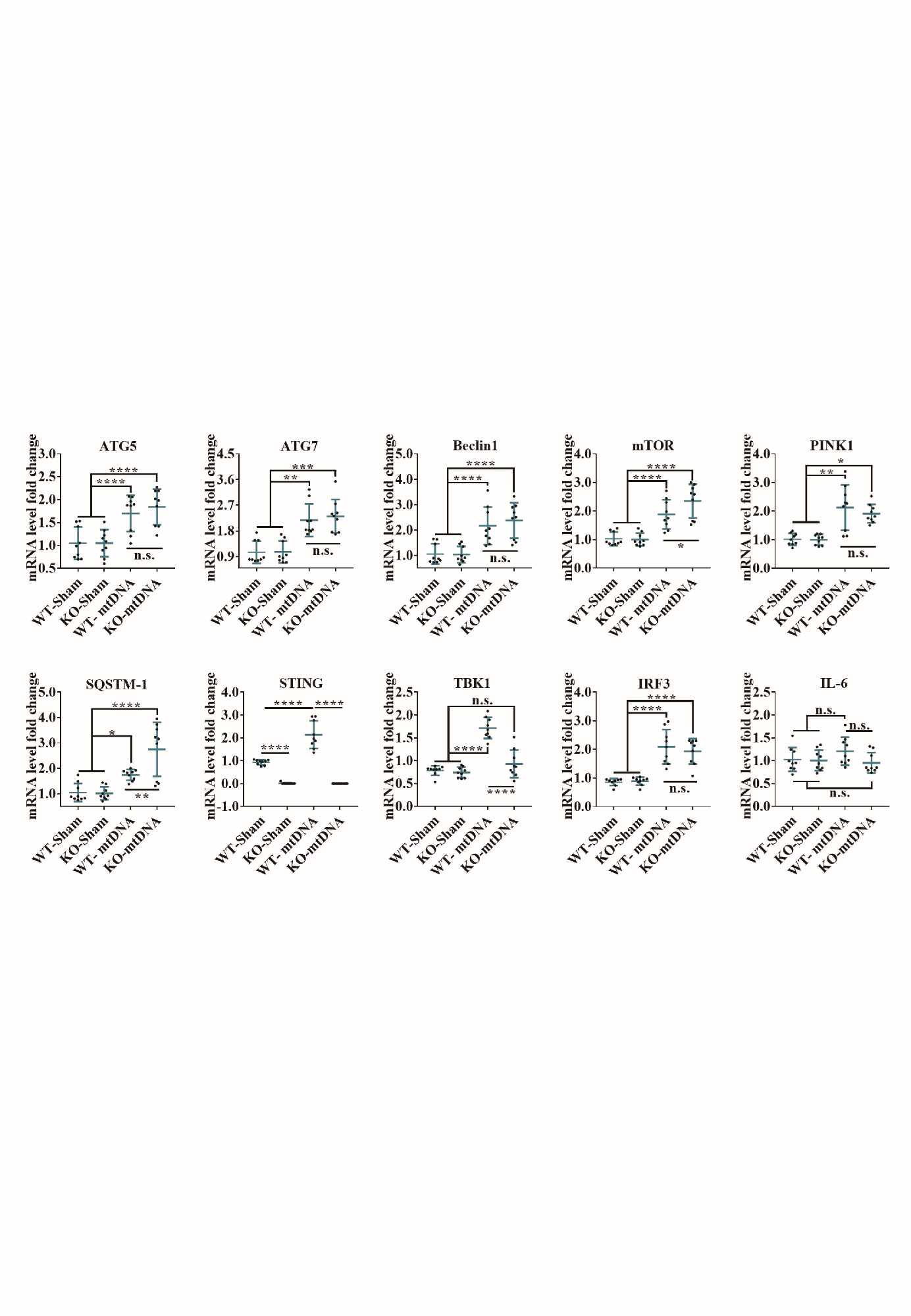


Figure S2. qPCR analysis of autophagy and STING signaling mRNA in the lung of WT and STING^-/-^ mice at 24h after mtDNA injection. Each panel shown represents the mean±SD. *p<0.05; **p<0.005; ***p<0.0001. ns, not signiﬁcant. Two-tailed Student’s t-test was used to determine statistical signiﬁcance


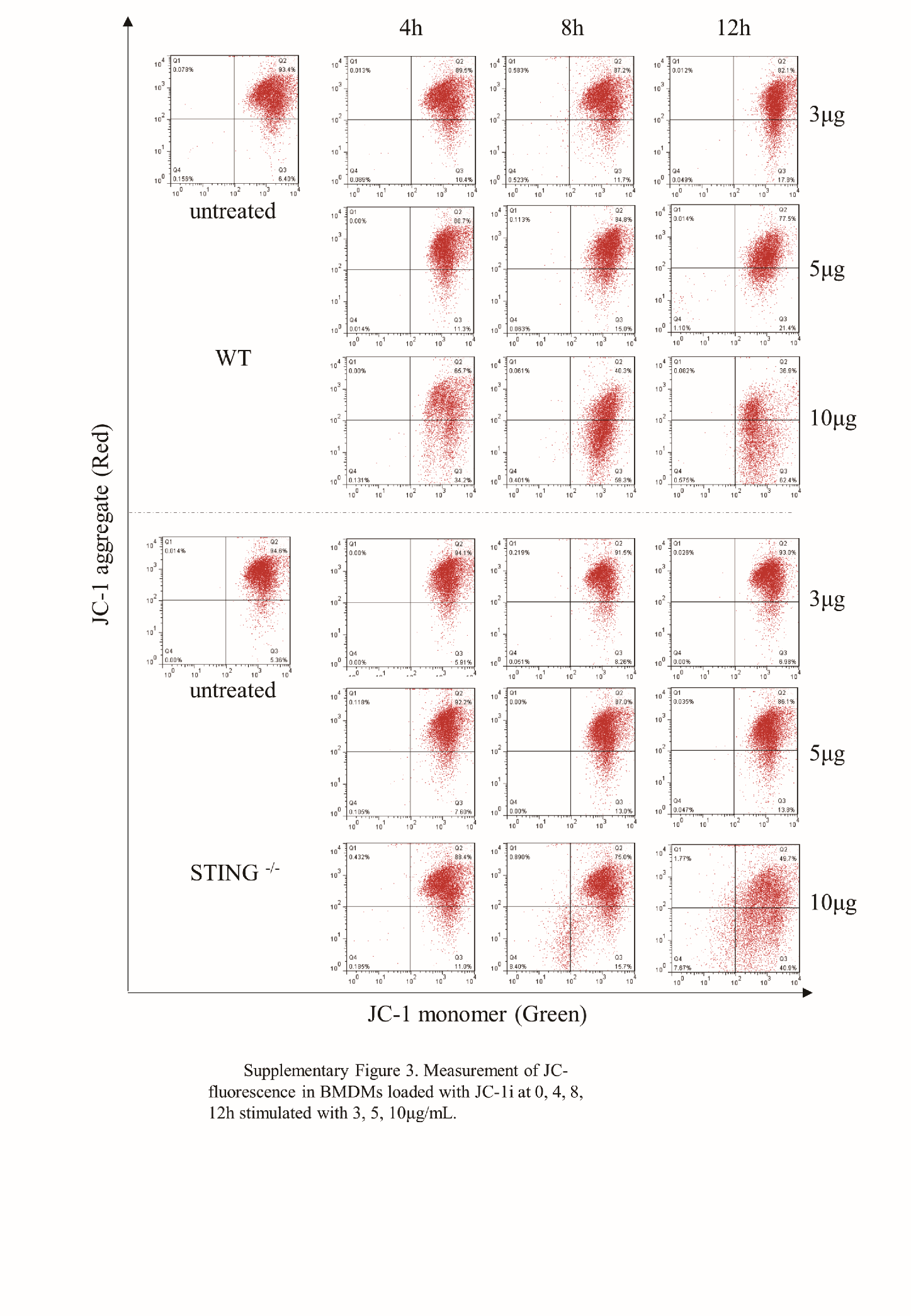


Figure S3. Representative images of JC-fluorescence in BMDMs loaded with JC-1 at 0, 4, 8, 12h stimulated with 3, 5, 10μg/mL.


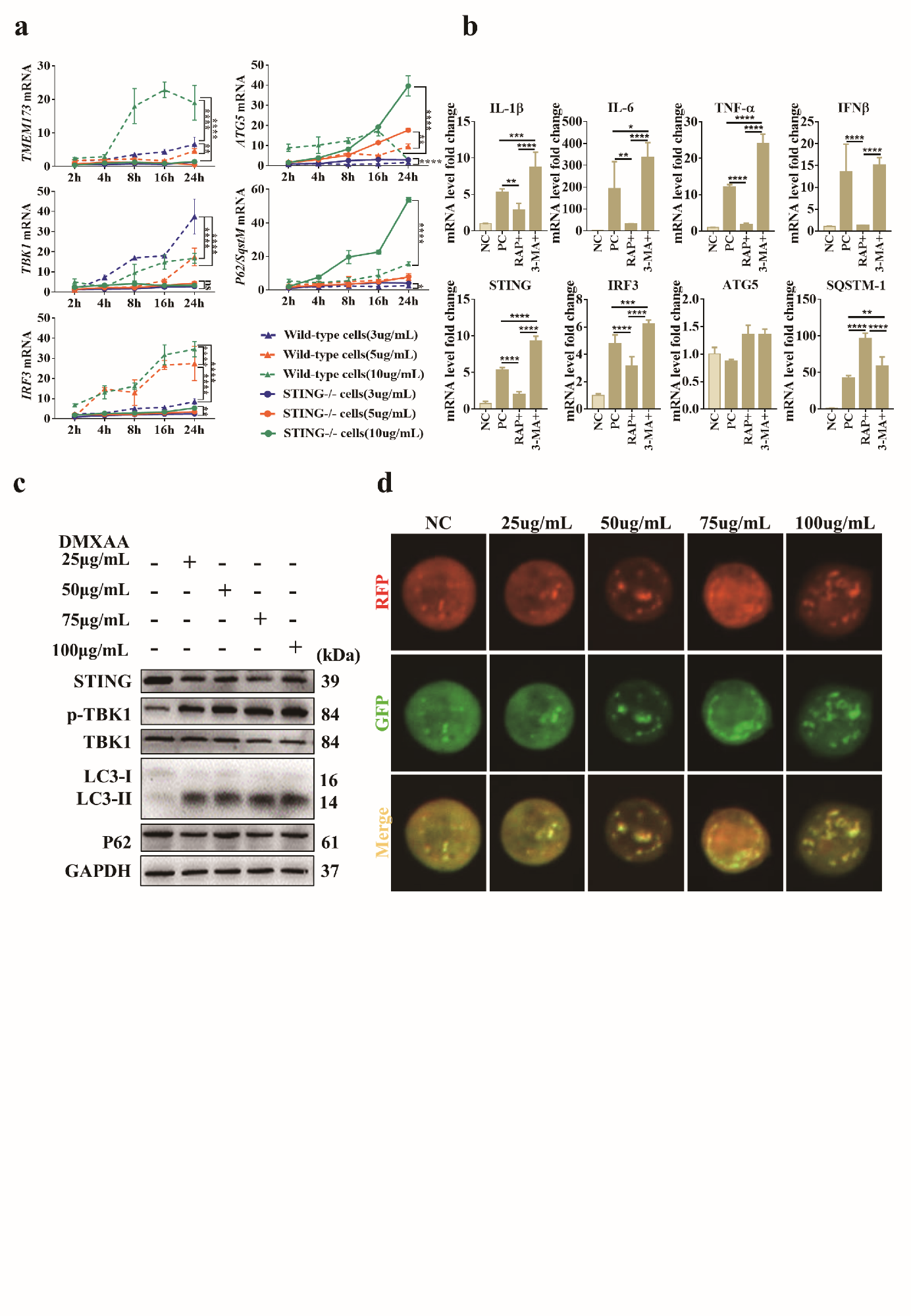


Figure S4. mtDNA and DMXAA induce STING-dependent impairments of autophagy. (a) qPCR analysis of STING and autophagy signaling mRNA in BMDMs after stimulated with 3,5, 10μg/mL mtDNA at 2, 4, 8, 16, 24h (n=3/each group). The levels of mRNA fold change were analyzed by two-tailed Student’s t-test was used to determine statistical signiﬁcance, *p<0.05; **p<0.005; ***p<0.0001. ns, not signiﬁcant. (b) RAW264.7 were pre-treated with RAP or 3-MA, and then stimulated with 75μg/mL DMXAA. The DMSO group and the DMXAA group were used as negative control (NC) and positive control (PC) (n=3/each group). The levels of mRNA fold change were analyzed by two-tailed Student’s t-test was used to determine statistical signiﬁcance, *p<0.05; **p<0.005; ***p<0.0001. ns, not signiﬁcant (n=3). (c) Western blot analysis of STING signaling and autophagy protein expression in RAW264.7 after stimulated with 25, 50, 75, 100μg/mL DMXAA at 3h. (d) Representative fluorescent images of RAW264.7 transfected with pMRX-IP-GFP-LC3-RFP-LC3ΔG and treated with 25, 50, 75, 100μg/mL DMXAA at 8h.


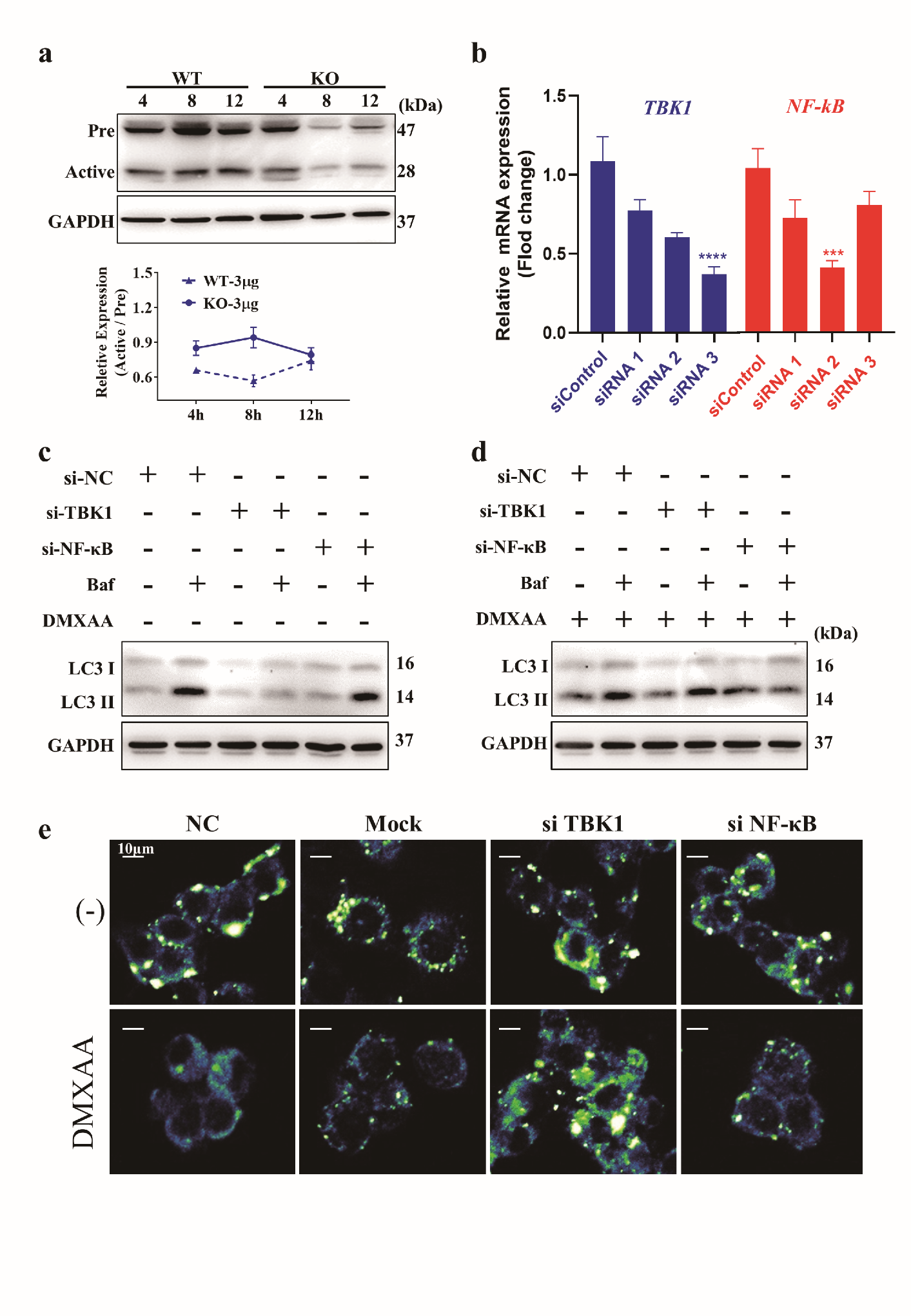


Figure S5. STING-mediated autophagic flux blockade is dependent on TBK1. (a) Western blot analysis of cathepsin D in BMDMs at 4, 8, 12h after stimulated with 3μg/mL mtDNA. (b) Verification of siTBK1s and siNF-κBs in RAW264.7 by qPCR (c-d) Western blot analysis of LC3 protein expression in RAW264.7 after stimulated with 75μg/mL DMXAA at 3h. Cells were transfected with control siRNA, TBK1 or NF-κB siRNA 24 h and pretreated with Baf or control before DMXAA treatment. Analyzed by two-tailed Student’s t-test was used to determine statistical signiﬁcance, *p<0.05; **p<0.005; ***p<0.0001. ns, not signiﬁcant. (e) Representative images of lysosome pH probe in RAW264.7. Cells were transfected with control siRNA or TBK1 siRNA 24 h before control or DMXAA treatment.


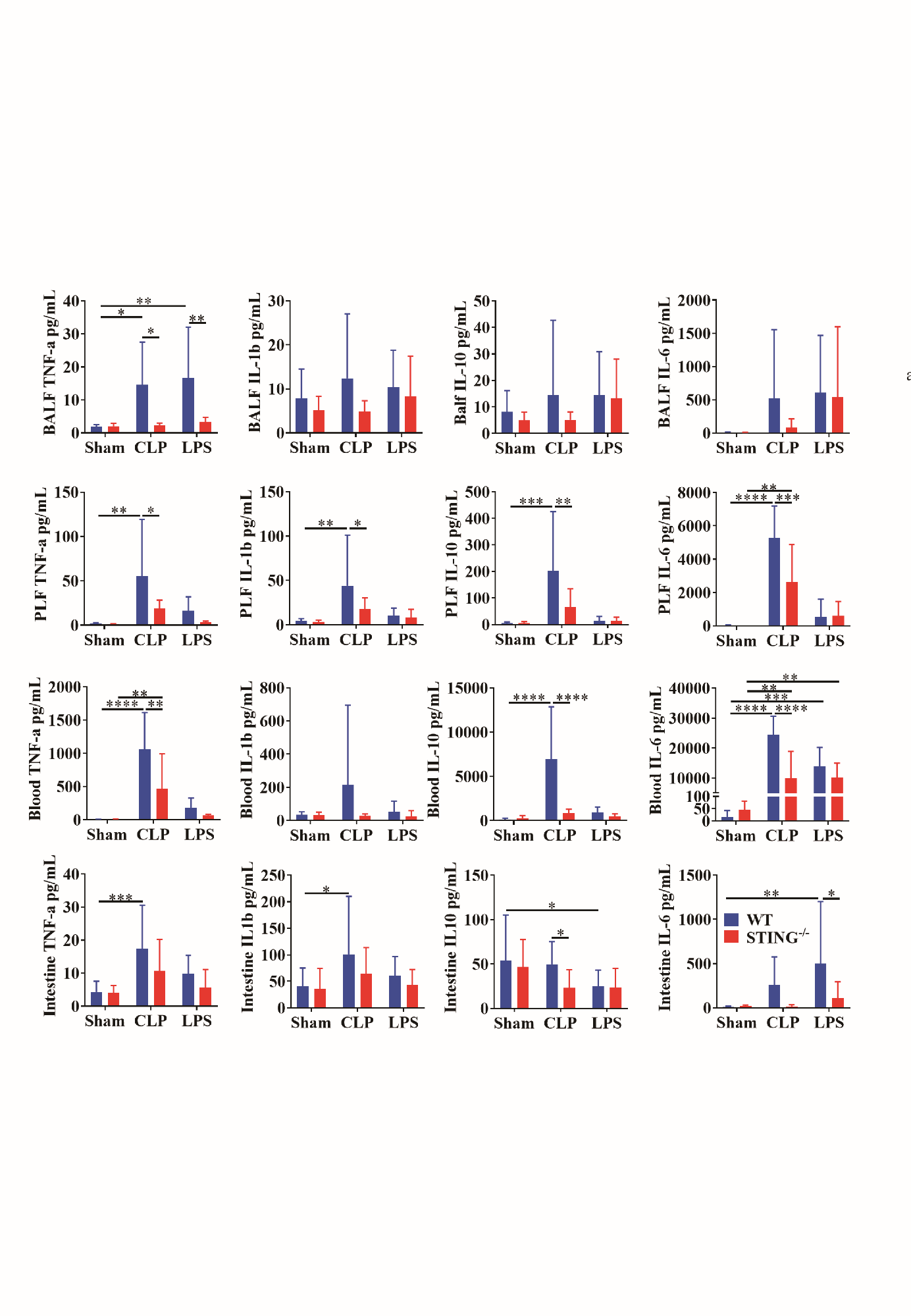


Figure S6. STING knockout protects against CLP and LPS systemic inflammation. BALF, PLF, plasma and intestine were collected form STING^-/-^ and WT group at 24h after subjected to CLP or LPS administration (n>4/each group). Analyzed by two-tailed Student’s t-test was used to determine statistical signiﬁcance, *p<0.05; **p<0.005; ***p<0.0001. ns, not signiﬁcant.

Table S1. Clinical characteristics of sepsis patients and sepsis-induced acute lung injury (sALI) individuals

|  | **Sepsis**  **N=12** | **sALI**  **N=11** | ***P* value** |
| --- | --- | --- | --- |
| **Age, years, Mean (SD)** | 34.17(11.167) | 54.27(16.953) | **0.003** |
| **Gender, male (%)** | 83.3 | 54.5 | 0.193 |
| **SOFA score, Mean (SD)** | 11.00(6.194) | 14.45(6.548) | **0.003** |
| **APACHE II score, Mean (SD)** | 7.42(4.033) | 12.00(5.177) | 0.208 |
| **Length of ICU, days, Mean (SD)** | 14.17(6.162) | 18.55(11.953) | 0.276 |
| **MV, days, Mean (SD)** | 0.00(0.000) | 13.45(13.232) | **0.007** |
| **28-day motality, (%)** | 8.3 | 63.6 | **0.009** |
